# CO-DESIGNING A CULTURALLY INFORMED DEMENTIA SYMPTOM SELF-MANAGEMENT MHEALTH APP WITH CARE PARTNERS

**DOI:** 10.1093/geroni/igad104.0461

**Published:** 2023-12-21

**Authors:** Aditi Durga, Shih-Yin Lin, Daniel David, Karen Moss, Meghan Reading Turchioe, Rupa Valdez, Abraham Brody

**Affiliations:** NYU Rory Meyers College of Nursing, New York City, New York, United States; NYU Rory Meyers College of Nursing, New York City, New York, United States; NYU Rory Meyers College of Nursing, New York City, New York, United States; The Ohio State University, Columbus, Ohio, United States; Columbia University, New York City, New York, United States; University of Virginia, Charlottesville, Virginia, United States; NYU Rory Meyers College of Nursing, New York City, New York, United States

## Abstract

Over 16 million care partners (CPs) in the U.S. provide more than 17 billion hours of unpaid care for family and friends living with dementia. Yet, significant inequities and access issues exist in assisting CPs to receive high quality support. Thus, this study aimed to tailor our existing evidence-based Aliviado Dementia Care mHealth app for diverse CP users through a user-centered approach to empower CPs with culturally informed knowledge, structure and resources. Based on needs identified by CPs, wireframing was performed. CPs then reviewed the interactive wireframes, were asked to perform various tasks, and provide feedback on any pain points, confusion, usability, and new ideas (“Think aloud”). Eight CPs (3 Black, 1 Asian, 4 White, 1 Hispanic; 38% Female; 29-77 years) from 6 different US states participated in the user testing. CPs found the wireframes easy to navigate and intuitive. Based on CP feedback, changes were made to include inclusive language, reconstruct the dashboard, and add medication alerts and a summary care profile. CPs voiced pleasant surprise that the app was going to have such a substantial feature set, including guiding them through person-centered assessment and care planning of interventions to non-pharmacologically self-manage behavioral and psychological symptoms of dementia and caregiving stress. We thus successfully developed an initial set of wireframes using co-design principles with CPs. CPs appreciated the goal of self-management and activation to work with their primary care providers. Future work will seek to test a functional model with diverse CPs in diverse participants in the community.

